# Identification and Detection of Aflatoxigenic *Aspergillus flavus* and Aflatoxins: Current Technologies and Future Perspectives

**DOI:** 10.3390/toxins18080343

**Published:** 2026-08-04

**Authors:** Md Mostafa Masud, Sumyya Waliullah, Abdus Sobhan, Emran Ali

**Affiliations:** 1 Department of Agriculture, Alcorn State University, Lorman, MS 39096, USA; 2 Department of Biological Sciences, Alcorn State University, Lorman, MS 39096, USA

**Keywords:** aflatoxigenic *Aspergillus flavus*, aflatoxins detection, identification, biosynthesis gene cluster, aflatoxins

## Abstract

*Aspergillus flavus* is a major fungal species that contaminates a wide range of crops and produces aflatoxins under favorable environmental conditions. Several approaches have been developed for aflatoxigenic *A. flavus* identification and aflatoxins detection. This review summarizes the existing methods for the identification of aflatoxigenic *A. flavus*, along with recent progress in aflatoxins detection methods. It also highlights limitations and possible factors associated with the accurate identification of aflatoxigenic isolates. Particularly, this review underscores the utilization of advanced analytical or detection methods along with existing identification techniques to accurately identify aflatoxigenic *A. flavus*.

## 1. Literature Search Strategy

A comprehensive search was performed across different electronic databases such as Google Scholar, PubMed, Web of Science, and ScienceDirect. The search included the published literature between 2007 and 2024, with a focus on existing methods for identification of aflatoxigenic *A. flavus*. Also, the literature included recent detection methods for aflatoxin quantification, particularly highlighting published articles between 2018 and 2025. Boolean operators were utilized to find the articles in the following terms: (“*Aspergillus flavus*” OR “*A. flavus*”) AND (aflatoxigenic OR toxigenic) AND (identification), (“*Aspergillus flavus*” OR “*A. flavus*”) AND (“aflatoxigenic” OR “toxigenic”) AND (“identification”) AND (“PCR” OR “RT-PCR” OR “RT-qPCR” OR “real-time” OR “LAMP”), ((“*Aspergillus flavus*” OR “*A. flavus*”) AND “toxigenic” AND “molecular” AND “detection”)), (“aflatoxin detection” OR “prediction” OR “mycotoxin detection”) AND (“biosensor” OR “artificial intelligence” OR “machine learning”), (“mycotoxin detection” AND “recent advances” OR “recent progress”).

## 2. Introduction

*Aspergillus flavus* is a major fungal contaminant of food and feed commodities, posing significant risks to both human and animal health. As an opportunistic and airborne pathogen, it readily infects agricultural products, particularly grains and nuts, under warm and humid environmental conditions. Consequently, *A. flavus* has emerged as a global concern, especially in tropical, subtropical, and Mediterranean regions where climatic conditions favor fungal growth and toxin production [1].

This pathogen can contaminate crops at multiple stages of the food chain, including pre-harvest, post-harvest, storage, and transportation [2,3]. A key concern associated with *A. flavus* is its ability to produce aflatoxins (AFs), a group of highly toxic secondary metabolites that compromise food safety and quality [4]. Among them, aflatoxin B1 (AFB1) is the most potent and is classified as a Group 1 carcinogen by the International Agency for Research on Cancer (IARC) [5]. In addition to AFB1, *A. flavus* produces AFB2 and other secondary metabolites such as cyclopiazonic acid. In contrast, AFM1 is not directly produced by the fungus but is a hydroxylated metabolite of AFB1 formed in animals such as poultry and livestock and commonly detected in milk following ingestion of contaminated feed [6].

AFs contamination is associated with substantial economic losses worldwide. In the United States alone, annual losses in the corn industry due to AFs contamination range from millions to billions of dollars [7]. Beyond economic impacts, AFs pose serious health risks, including hepatotoxicity, immunosuppression, and hepatocellular carcinoma, particularly in individuals co-infected with hepatitis B virus [6,8,9,10,11,12,13,14,15,16,17,18]. Therefore, effective monitoring and accurate detection of aflatoxigenic *A. flavus* are critical for ensuring food safety and mitigating public health risks.

Traditionally, identification of *A. flavus* has relied on morphological and cultural characteristics, including colony morphology, pigmentation, and microscopic features [19,20]. However, these methods are labor-intensive, time-consuming, and often lack the sensitivity and specificity required to distinguish between aflatoxigenic and atoxigenic strains [21,22]. Moreover, there are no reliable morphological markers that can differentiate aflatoxigenic isolates from non-aflatoxigenic ones.

To overcome these limitations, molecular approaches have been widely adopted for the detection and characterization of aflatoxigenic *A. flavus*. These methods typically target genes within the AFs biosynthesis gene cluster, which is responsible for toxin production. Although molecular techniques such as PCR and sequencing offer higher sensitivity and specificity [21,23,24,25], challenges remain due to genetic variability, the absence or mutation of specific genes, and the inconsistent relationship between gene presence, gene expression, and actual AFs production [26,27,28,29].

This review mainly focuses on the traditional and existing methods that have been widely used for the identification of aflatoxigenic *A. flavus* and highlights the potential limitations associated with this method. Even though an isolate of *A. flavus* is identified through morphological and molecular methods, it cannot produce AFs in real time. Therefore, this review also highlights how the identification technique could be well adapted along with the AFs detection technique to accurately predict the aflatoxigenic *A. flavus* isolates. Moreover, several factors need to be considered while measuring AFs detection because they could influence the final concentration of AFs accumulation in food and feed crops.

Therefore, a comprehensive understanding of existing detection methods, along with their limitations, is essential for developing reliable and efficient diagnostic tools. It also emphasizes the need for integrated, multi-method strategies to enhance the accuracy and reliability of detection in both laboratory and field settings.

## 3. Traditional Approaches/Cultural Techniques

Morphological identification is one of the earliest and most widely used approaches for detecting fungal pathogens, including *A. flavus*. This method relies on observable characteristics such as colony morphology, growth pattern, pigment production, hyphal structure, and conidial formation on culture media [30,31,32,33,34]. Based on these traits, *A. flavus* isolates can be preliminarily identified by culturing on different types of media, each designed to support specific growth or analytical objectives (Table 1).

Different culture media are selected depending on the purpose of the study, including isolation, identification, toxin production assessment, and gene expression analysis. As summarized in Table 1, media such as *A. flavus* and *parasiticus* agar (AFPA), Aflatoxins-producing ability medium (APA), coconut-based media (CMA, CEA, CCA), Czapek yeast extract agar (CYA), and yeast extract sucrose (YES) media are widely used for culturing *Aspergillus* section Flavi under varying experimental conditions [35].

Despite its widespread use, morphological and cultural identification has several limitations. These methods require considerable time for media preparation, fungal growth, and microscopic examination. Additionally, accurate identification often requires substantial mycological expertise. More importantly, morphological approaches lack reliability due to significant intraspecific and interspecific variability among *Aspergillus* species [36]. As a result, distinguishing aflatoxigenic and non-aflatoxigenic strains based solely on morphology is not feasible.

To address these limitations, several culture-based techniques have been developed to identify aflatoxigenic and non-aflatoxigenic isolates based on their ability to produce AFs. These include the ammonia vapor test and fluorescence-based detection under UV light, which are discussed below.

**Table 1:** Different types of media that have been utilized by several researchers for culturing *Aspergillus* section *Flavi*.

Media	Application	References
AFPA	Selective media for the visual detection of *A. flavus* and *A. parasiticus*, Visual differentiation of aflatoxigenic and non-aflatoxigenic *A. flavus*	[16,37,38]
CYA, YES, CAM, APA, CCA, CMA, AFPA	Useful for growing aflatoxigenic *A. flavus* and determining AFs production capability.	[39,40,41,42,43]
DRBC, MRBA	Isolation and identification of *Aspergillus* spp. from soil	[16,44,45]
YESD	Better visual identification of aflatoxigenic *A. flavus* strains	[38]
MEA, PDA, YES, CYA	Visualization of the differences in morphological characteristics in *A. flavus* and *A. oryzae*, such as colony color, growth rate, sporulation, and formation of conidial chain, vesicle formation, and shape of conidia	[46]
PDA	Widely used media for visualization of macroscopic and microscopic structures of *A. flavus*, Culture-based detection methods based on color change, such as the Ammonia Vapor test and Fluorescence-based detection.	[42,47,48,49]
PDB and Modified PDB	Widely used for the higher growth of *Aspergillus* spp. to perform DNA extraction and measure AFs production.	[50,51,52,53,54]
MACM	Differences in AFB1 production by *A. flavus* through several sampling time periods and *A. flavus* growth over time and its dynamics.	[55]
PNA	To observe the interaction of climate change-related abiotic factors (temperature and water stress) on growth, gene expression, and AFB1 production	[56]

Note: AFPA: *A. flavus* and *parasiticus* agar; APA: Aflatoxins producing ability medium; CMA: Coconut milk agar; CEA: coconut extract agar; CCA: coconut cream agar; PDA: Potato Dextrose Agar; PDB: Potato Dextrose Broth; CYA: Czapek yeast extract agar; YESD: Yeast extract sucrose agar supplemented with methyl β-cyclodextrin and sodium desoxycholate; MACM: Maize Agar Culture Medium; PNA: Pistachio Nut Agar; YES: Yeast extract sucrose broth; MRBA: Modified Rose Bengal Agar Media; DRBC: Dichloran rose bengal chloramphenicol agar.

### 3.1. Ammonia Vapor Test

The ammonia vapor test is a conventional, rapid, and cost-effective method used to differentiate aflatoxigenic and non-aflatoxigenic *A. flavus* isolates. This technique is particularly useful for preliminary screening of non-aflatoxigenic strains for potential use as biocontrol agents.

**Figure 1:**
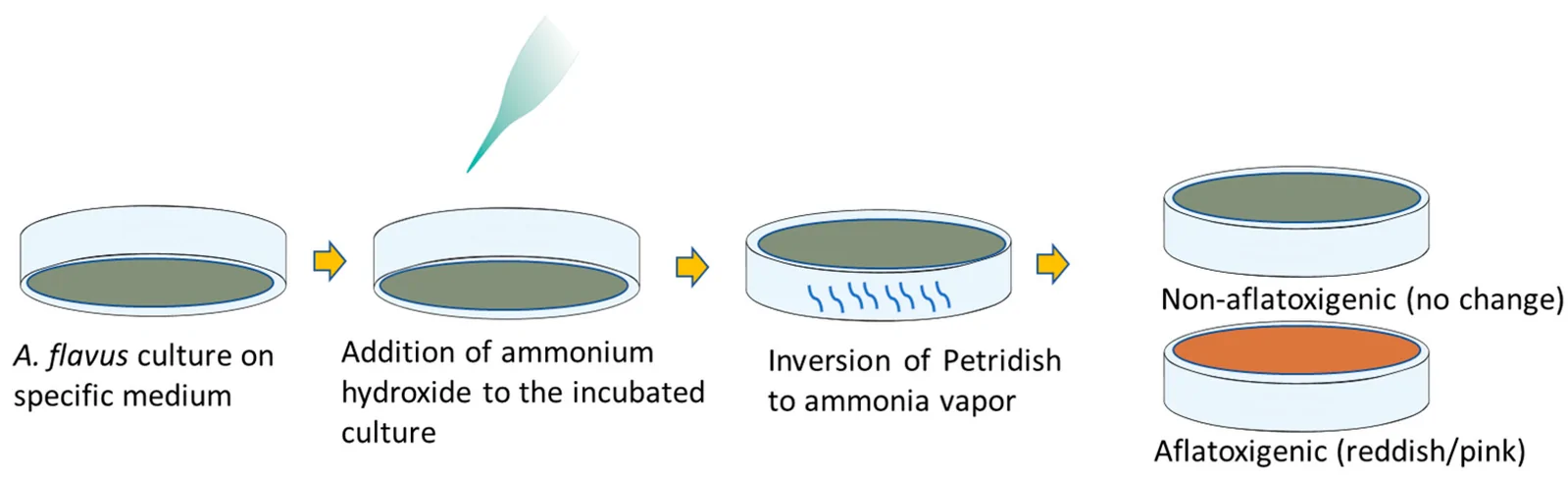
Differentiation of aflatoxigenic and non-aflatoxigenic *A. flavus* isolates using the ammonia vapor test. Exposure to ammonium hydroxide vapor induces color changes in colonies due to pH-sensitive AFs-associated pigments, enabling visual discrimination of aflatoxigenic isolates.

In this method, *A. flavus* isolates are cultured on suitable agar media and exposed to ammonium hydroxide vapor by placing a drop of ammonia solution on the lid of an inverted Petri dish. After exposure, color changes in the fungal colonies are observed, which are associated with AFs production (Figure 1). The color variation is attributed to the interaction between ammonia vapor and AFs-related pigments, including averantin, norsolorinic acid, averufin, versicolorin A, versicolorin C, nidurufin, and versicolorin A hemiacetal [57]. These pigments act as pH-sensitive indicators, changing color in response to increased pH conditions.

Several studies have demonstrated the application of this method. For example, ref. [19] evaluated 25 *A. flavus* isolates and successfully differentiated aflatoxigenic and non-aflatoxigenic strains based on color variation. Similarly, ref. [20] reported that aflatoxigenic isolates produced a reddish-pink coloration on coconut agar medium (CAM) following ammonia treatment. The dichlorvos-ammonia (DV-AM) method has also been employed, where isolates grown on YES-DOC-CP-DV medium exhibited a yellow-to-red color change upon ammonia exposure [58].

Despite its advantages, the ammonia vapor test has several limitations. It is a qualitative method that does not provide quantitative information on AFs levels. Additionally, results may vary depending on culture age, media composition, pigment availability, and environmental conditions [56]. Therefore, this method is not standardized and should be used primarily as a preliminary screening tool rather than a definitive diagnostic approach.

### 3.2. Fluorescent-Based Detection

Fluorescence-based detection is another widely used culture-dependent method for identifying aflatoxigenic *A. flavus*. This technique relies on the emission of blue to blue-green fluorescence when fungal cultures are exposed to UV light (365 nm) on both solid and liquid media such as potato dextrose agar (PDA), coconut agar medium (CAM), and aflatoxins-producing ability (APA) medium [59].

**Figure 2:**
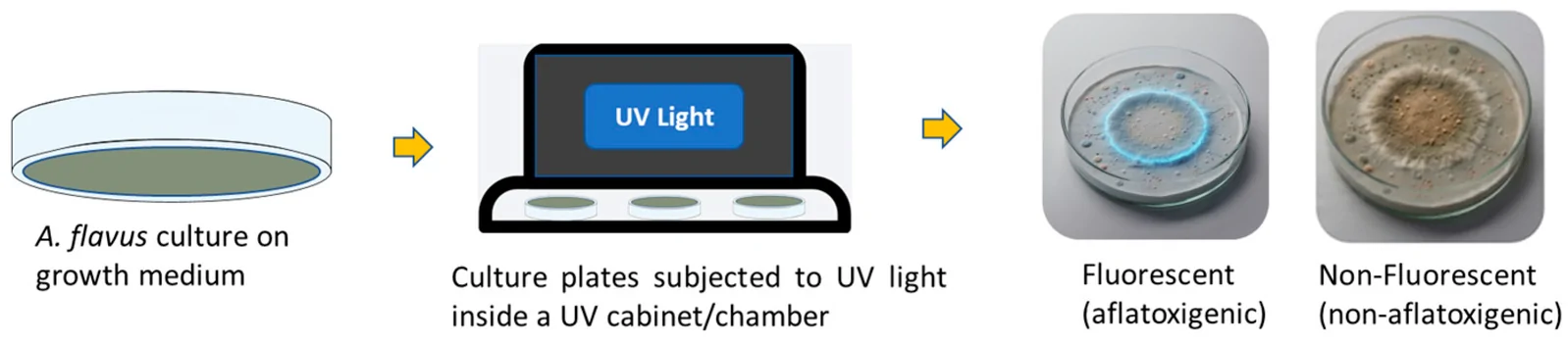
Fluorescence-based detection of aflatoxigenic *A. flavus* isolates under UV light (365 nm). Aflatoxigenic strains exhibit blue to blue-green fluorescence due to AFs production, whereas non-aflatoxigenic isolates show little or no fluorescence.

In this method, fungal colonies are incubated and then observed under UV light. Aflatoxigenic isolates typically produce fluorescence, whereas non-aflatoxigenic isolates do not [57,60] (Figure 2). For example, ref. [16] demonstrated fluorescence-based detection using coconut cream agar (CCA), where enhanced visualization was achieved through the addition of chloramphenicol and incubation under controlled conditions.

The intensity of fluorescence can be enhanced using additives such as cyclodextrins and iodine, which improve AFs visualization [61]. Comparative studies have shown varying detection efficiencies; for instance, ref. [62] reported that 62% of isolates tested positive using UV fluorescence, whereas 54% were identified using the ammonia vapor test.

Although fluorescence-based detection is relatively simple and cost-effective, it also has limitations. Fluorescence intensity can vary depending on media composition, environmental conditions, and fungal growth stage. Furthermore, these methods do not provide quantitative data and may lead to misclassification of isolates.

### 3.3. Limitations of Cultural Methods and Need for Molecular Approaches

Overall, cultural methods are advantageous due to their low cost and simplicity; however, they lack sensitivity, specificity, and reproducibility. AFs production is influenced by multiple factors, including nutrient availability, pH, temperature, oxidative stress, and environmental conditions [63]. For example, low-nutrient media may reduce fungal growth and subsequently limit AFs production [63,64].

Therefore, while cultural techniques are useful for preliminary screening, they are insufficient for accurate identification of aflatoxigenic *A. flavus*. This limitation highlights the need for molecular approaches, which provide greater precision by targeting genes involved in AFs biosynthesis.

## 4. AFs Biosynthesis Gene Cluster

The AFs biosynthesis gene cluster is a complex genomic region approximately 70 kb in size, located near the telomeric region of chromosome 3 in *A. flavus* [63,65]. This cluster contains a large number of structural and regulatory genes that collectively encode enzymes involved in the AFs biosynthesis pathway (Table 2).

At least 27 enzymatic reactions are involved in AFs production, forming a highly coordinated polyketide pathway [66]. The expression of these genes is primarily regulated by two key transcriptional regulators, *aflR* and *aflS* [63,67]. Among these, *aflR* acts as a major transcriptional activator belonging to the *Gal4* family and plays a central role in regulating the expression of downstream biosynthetic genes [68]. Overexpression of *aflR* has been shown to enhance transcriptional activity and promote AFs production [69].

The *aflS* gene functions as a co-activator that enhances the regulatory activity of *aflR*. Interestingly, studies have shown that *aflS* expression is more closely associated with AFs production than *aflR*, as *aflS* expression decreases under conditions where AFs production is reduced, even when *aflR* expression remains stable [70]. This suggests that *aflS* may play a critical role in fine-tuning the regulation of AFs biosynthesis.

In addition to these regulatory genes, several transcription factors such as bZIP proteins (e.g., *atfA* and *atfB*) also influence AFs production by responding to oxidative stress and environmental signals. These factors bind to gene promoters and regulate transcriptional responses under stress conditions.

### 4.1. Stages of AFs Biosynthesis

**Figure 3:**
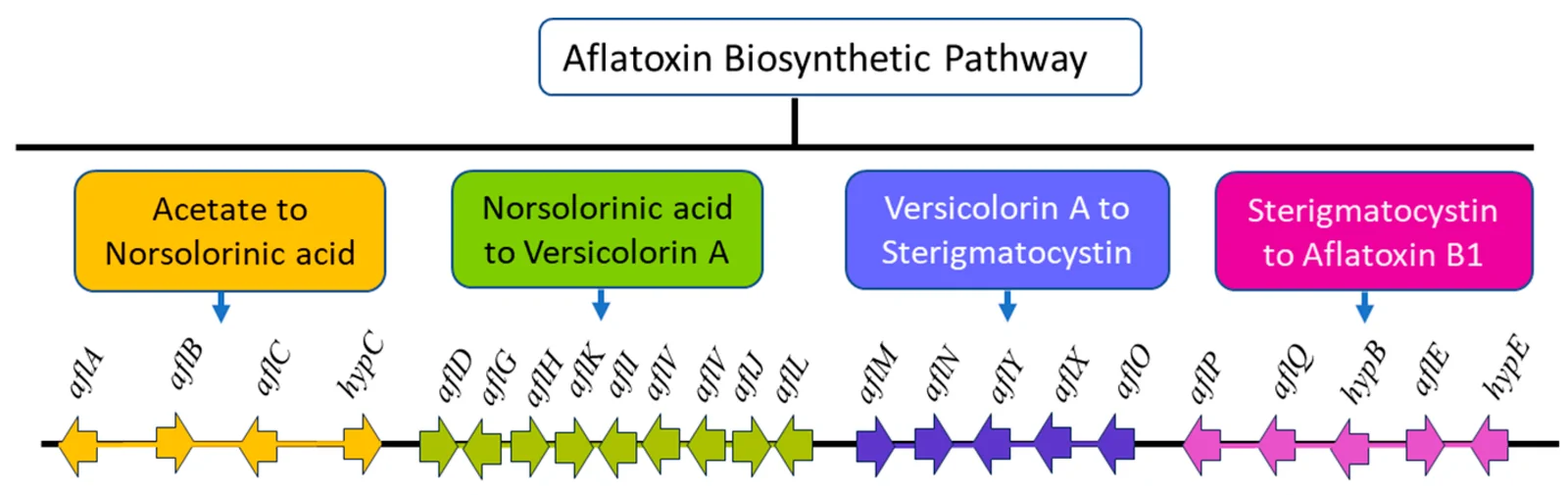
Schematic representation of the AFs biosynthesis pathway in *A. flavus*, illustrating key intermediate compounds and associated genes involved in AFB1 production.

The AFs biosynthesis pathway can be divided into four major stages based on the formation of intermediate compounds (Figure 3):

#### 4.1.1. Stage 1: Formation of Norsolorinic Acid (NOR)

The initial stage involves the conversion of acetate into norsolorinic acid (NOR), the first stable intermediate. This process is mediated by genes such as *aflA*, *aflB*, *aflC*, and *hypC* [71,72].

#### 4.1.2. Stage 2: Conversion to Versicolorin A (VERA)

In this stage, NOR is converted into versicolorin A through a series of enzymatic reactions involving genes such as *aflD*, *aflG*, *aflH*, *aflK*, *aflI*, *aflV*, *aflW*, *aflJ*, and *aflL* [65,73,74,75,76,77,78,79,80,81].

#### 4.1.3. Stage 3: Formation of Sterigmatocystin (ST)

Versicolorin A is further converted into sterigmatocystin through the action of genes such as *aflM*, *aflN*, *aflY*, *aflX*, and *aflO* [79,82,83,84].

#### 4.1.4. Stage 4: Final Conversion to Aflatoxin B1 (AFB1)

The final stage involves the conversion of sterigmatocystin into aflatoxin B1 through genes such as *aflP*, *aflQ*, *hypB*, *aflE*, and *hypE* [24,85,86,87].

Among these stages, genes involved in the final steps of the pathway are considered particularly important, as they directly contribute to AFs production.

### 4.2. Regulation and Environmental Influence

AFs biosynthesis is not solely determined by gene presence but is strongly influenced by environmental and physiological factors. Variables such as temperature, CO_2_ concentration, nutrient availability, oxidative stress, and developmental stage significantly affect gene expression and toxin production [88,89].

For example, nutrient-rich conditions promote AFs biosynthesis; whereas, nutrient limitation suppresses production. Similarly, oxidative stress plays a key role in regulating gene expression through transcription factors such as *atfA* and *atfB*.

### 4.3. Implications for Detection

Although the presence of AFs biosynthesis genes is often used as a marker for identifying aflatoxigenic isolates, it does not guarantee toxin production. The complexity of gene regulation, combined with environmental influences, makes it difficult to accurately predict AFs production based solely on gene presence.

Therefore, effective identification of aflatoxigenic *A. flavus* requires an integrated approach that considers gene presence, gene expression, and environmental factors. This complexity underscores the importance of combining molecular and analytical methods for reliable identification.

**Table 2:** AFs biosynthesis genes, their encoded enzymes, and associated functions. This table is adapted from [63,65,81,87,90].

Updated and Previous Name	Encoded Enzyme and Function	Stage of AFs Biosynthesis
*aflA* (*fas-2*)	Fatty acid synthase α; conversion of hexanoate units into a polyketide structure	Conversion of acetate to norsolorinic acid (NOR)
*aflB* (*fas-1*)	Fatty acid synthase β; conversion of hexanoate units into a polyketide structure	Conversion of acetate to NOR
*aflC* (*pksA*)	Polyketide synthase; synthesis of polyketide skeleton and conversion into norsolorinic acid anthrone (NAA)	Conversion of acetate to NOR
*hypC* (*hypB1*)	Anthrone oxidase; conversion of NAA into NOR	Conversion of acetate to NOR
*aflD* (*nor-1*)	Norsolorinic acid ketoreductase; conversion of NOR to averantin (AVN)	Conversion of NOR to versicolorin A
*aflG* (*avnA*)	Cytochrome P450 monooxygenase; hydroxylation of averantin into 5′-hydroxyaverantin (HAVN)	Conversion of NOR to versicolorin A
*aflH* (*adhA*)	Alcohol dehydrogenase; conversion of HAVN into 5′-oxoaverantin (OAVN)	Conversion of NOR to versicolorin A
*aflK* (*vbs*)	Cyclase/VERB synthase; transformation of versiconal into versicolorin B and conversion of intermediates to versicolorin B	Conversion of NOR to versicolorin A
*aflI* (*avfA*)	Averufin oxidase; conversion of averufin (AVF) into versiconal hemiacetal acetate (VHA)	Conversion of NOR to versicolorin A
*aflV* (*cypX*)	Cytochrome P450 monooxygenase; conversion of AVF into hydroxyversicolorone (HVN) and conversion of HVN into VHA	Conversion of NOR to versicolorin A
*aflJ* (*estA*)	Esterase; conversion of VHA into versiconal (VAL)	Conversion of NOR to versicolorin A
*aflL* (*verB*)	Cytochrome P450 monooxygenase/desaturase; conversion of versicolorin B into versicolorin A (VERA)	Conversion of NOR to versicolorin A
*aflM* (*ver-1*)	Ketoreductase; conversion of versicolorin A into demethylsterigmatocystin (DMST)	Conversion of versicolorin A to sterigmatocystin
*aflN* (*verA*)	Cytochrome P450 monooxygenase; conversion of versicolorin A into intermediate compounds	Conversion of versicolorin A to sterigmatocystin
*aflY* (*hypA*)	Hypothetical protein; involved in conversion of VERA to DMST	Conversion of versicolorin A to sterigmatocystin
*aflX* (*ordB*)	Oxidoreductase; catalyzes oxidative decarboxylation and ring closure reactions	Conversion of versicolorin A to sterigmatocystin
*aflO* (*omtB*)	O-methyltransferase; conversion of DMST into sterigmatocystin (ST)	Conversion of versicolorin A to sterigmatocystin
*aflP* (*omtA*)	O-methyltransferase; conversion of sterigmatocystin into O-methylsterigmatocystin (OMST)	Conversion of sterigmatocystin to AFB1
*aflQ* (*ordA*)	Cytochrome P450 monooxygenase; conversion of OMST to AFB1 and G1	Conversion of sterigmatocystin to AFB1
*aflE* (*norA*)	Aryl alcohol dehydrogenase; involved in late-stage AFs biosynthesis	Conversion of sterigmatocystin to AFB1
*hypE* (*aflLa*)	Hypothetical protein; involved in final steps of AFs production	Final stage of AFs biosynthesis

Note: NOR = norsolorinic acid; AVN = averantin; HAVN = hydroxyaverantin; OAVN = oxoaverantin; AVF = averufin; VHA = versiconal hemiacetal acetate; VAL = versiconal; VERA = versicolorin A; DMST = demethylsterigmatocystin; ST = sterigmatocystin; OMST = O-methylsterigmatocystin.

## 5. Existing Molecular Methods and Potential Drawbacks

Molecular detection approaches have become indispensable tools for identifying aflatoxigenic *A. flavus*, particularly due to the limitations associated with conventional morphological and cultural methods. As previously discussed, traditional diagnostic techniques, including visual symptom assessment, culturing, and biochemical assays, are often labor-intensive, time-consuming, and lack sufficient sensitivity and specificity [91].

To address these challenges, molecular techniques such as PCR, real-time PCR (qPCR), reverse transcription PCR (RT-PCR), multiplex PCR, loop-mediated isothermal amplification (LAMP) have been widely adopted [92,93] (Table 3). These approaches enable rapid, sensitive, and specific detection of *A. flavus*, even in complex environmental and food matrices. However, despite these advancements, accurate classification of aflatoxigenic isolates remains difficult due to variability in gene presence, gene expression, and environmental influences on AFs biosynthesis.

As shown in Table 4, multiple molecular methods are currently employed to detect aflatoxigenic *A. flavus* by targeting genes from the AFs biosynthesis gene cluster. While some molecular approaches improve identification efficiency, their ability to accurately classify isolates as aflatoxigenic or non-aflatoxigenic depends on integrating multiple layers of information, including gene presence, expression, and toxin quantification. Also, as shown in Table 3, no single method is sufficient for accurate identification of aflatoxigenic *A. flavus* because solely molecular detection could not identify aflatoxigenic isolates. Rather, integration of molecular and quantification methods provides better insights into aflatoxigenic *A. flavus* isolates.

**Table 4:** Use of several AFs biosynthesis genes and molecular methods to identify aflatoxigenic *A. flavus* in different crops, food and feed.

Molecular Method	Targeted Genes/Regions	Sample Source	Identification	References
Conventional PCR	Biosynthetic Pathway genes (*aflR*, *aflS aflD*, *aflP*, *aflR*, *aflD*, *aflP*, *aflR*, *aflM*, *aflR*, *aflS*, *aflQ*, *aflP*, *aflD*, *aflM*, *aflO*)	Food, poultry feed, grains, stored grains, nuts, peanuts, spices	aflatoxigenic *A. flavus*	[20,94,95,96,97,98,99,100,101]
	Barcoding regions (ITS1, ITS4, Species-specific primer, *CaM*)	Chicken liver		[102,103,104]
PCR-RFLP	*aflP*	Peanut	aflatoxigenic *A. flavus*	[105]
Multiplex PCR/Rt-PCR/qPCR	Biosynthetic Pathway Genes (*aflD*, *aflM*, *aflP*, *aflQ*, *aflD*, *aflO*, *aflQ*, *aflG*, *aflH*, *aflI*, *aflK*, *aflT*, *aflR*, *aflS*, *laeA*, *lipA*) and Housekeeping genes (*calmodulin*, *β-tub*)	Peanuts, soil and stored cultures, Brazil nuts, corn, almonds, hazelnuts, coffee, and pistachio nuts	aflatoxigenic *A. flavus*	[23,24,51,64,69,106,107,108,109,110,111,112,113]
LAMP Assay	Biosynthetic Pathway and Housekeeping genes (*aflP*, *aflD*, *aflT and acl1*)	Food samples, brazil nuts, spices, dried fruit, nuts and coffee	aflatoxigenic *A. flavus*	[30,114,115,116,117]

### 5.1. PCR

Polymerase chain reaction (PCR) is one of the most widely used molecular techniques for detecting *A. flavus*, based on the amplification of specific DNA fragments. The amplified products are typically visualized using gel electrophoresis with fluorescent dyes [118]. PCR offers several advantages, including rapid detection, high sensitivity, and the ability to analyze samples without extensive culturing.

Early applications of PCR in fungal identification focused on ribosomal DNA (rDNA) regions, particularly the internal transcribed spacer (ITS), which is highly variable and useful for species-level identification [119,120,121]. ITS-based assays have been successfully used to detect *A. flavus* and differentiate it from closely related species such as *A. parasiticus* [27,122,123]. In addition, protein-coding genes such as *calmodulin* (*CaM*) and *β-tubulin* (*BenA*) provide improved phylogenetic resolution within *Aspergillus* section Flavi [124,125].

For detecting aflatoxigenic strains, PCR assays often target genes within the AFs biosynthesis gene cluster, including regulatory genes (*aflR*, *aflT*) and structural genes (*aflD*, *aflM*, *aflP*) [108,126]. Several studies have demonstrated that combinations of these genes can be used to differentiate aflatoxigenic and non-aflatoxigenic isolates [20,127]. However, PCR-based methods have significant limitations. They primarily detect gene presence and do not provide information on gene expression or functional activity. For instance, the presence of *aflR* alone is insufficient to confirm AFs production, as it regulates multiple metabolic pathways [128].

Additionally, PCR assays are prone to false-positive and false-negative results due to primer mismatches, mutations within target genes, DNA quality, and low template concentration. Despite extensive research using various biosynthetic genes, no standardized gene set has been established for definitive identification of aflatoxigenic *A. flavus* [129,130]. Therefore, PCR alone is insufficient for accurate identification and must be complemented with other molecular or analytical techniques.

### 5.2. Multiplex PCR

Multiplex PCR enables simultaneous amplification of multiple target genes in a single reaction, providing a more comprehensive and efficient detection strategy compared to conventional PCR [113]. This method is particularly useful for targeting multiple genes from different stages of the AFs biosynthesis pathway, thereby improving detection reliability.

Several studies have utilized multiplex PCR to detect aflatoxigenic fungi using gene sets such as *nor-1*, *ver-1*, *omt-A*, and *apa-2* [108]. Similarly, multiplex PCR has been applied to detect multiple mycotoxigenic fungi across different genera, including *Aspergillus*, *Fusarium*, and *Penicillium* [113]. Other studies have targeted gene combinations such as *omtA*, *omtB*, *ver-1*, and *aflR* for detecting aflatoxigenic *A. flavus* isolates from environmental samples [107].

Despite its advantages, multiplex PCR has limitations related to primer interactions, which can reduce amplification efficiency and sensitivity [106]. More importantly, similar to conventional PCR, multiplex PCR does not account for gene expression or toxin production. Therefore, although it improves detection coverage, it cannot definitively identify isolates as aflatoxigenic.

### 5.3. Reverse Transcriptase Polymerase Chain Reaction (RT-PCR)

RT-PCR is a powerful technique used to evaluate gene expression by converting RNA into complementary DNA (cDNA) prior to amplification. This approach provides insights into gene activity rather than mere gene presence, making it particularly valuable for studying AFs biosynthesis pathways.

Several studies have reported correlations between the expression of specific genes and AFs production. For example, *aflQ* and *aflO* genes have consistently shown strong positive correlations with AFs accumulation [23,111,113]. Similarly, *aflD* has been identified as an early-stage gene involved in AFs biosynthesis [112,131].

However, gene expression patterns are highly variable and influenced by environmental conditions, growth media, incubation time, and nutrient availability [89,124,132]. In some cases, isolates expressing multiple biosynthetic genes fail to produce detectable AFs levels, resulting in false-positive interpretations [112]. Furthermore, genetic mutations and regulatory differences can affect transcriptional activity, complicating interpretation [124].

These inconsistencies highlight that although RT-PCR provides valuable insights into gene expression, it cannot independently confirm AFs production. Therefore, it should be integrated with quantitative and analytical methods for accurate detection.

### 5.4. Quantitative PCR (qPCR)

Quantitative PCR (qPCR), also known as real-time PCR, allows simultaneous detection and quantification of DNA targets using fluorescent dyes or probes. This technique is widely used for detecting *A. flavus* in food, feed, and environmental samples due to its high sensitivity and specificity [133,134,135,136].

Several studies have demonstrated the effectiveness of qPCR in detecting AFs biosynthesis genes such as *omt-1*, *aflT*, and *aflR*, with detection limits as low as nanogram to femtogram levels [134,137,138].

However, qPCR shares similar limitations with conventional PCR, as it primarily detects gene presence rather than functional toxin production. Mutations within the AFs biosynthesis gene cluster and variability in gene expression can lead to inaccurate identification [135]. Therefore, integrating qPCR with RT-PCR and toxin quantification methods enhances detection reliability.

### 5.5. LAMP (Loop-Mediated Isothermal Amplification) Assay

Loop-mediated isothermal amplification (LAMP) is a rapid and sensitive DNA amplification technique that operates under constant temperature conditions [139]. This eliminates the need for thermal cycling equipment, making it suitable for field-based applications.

Several LAMP assays have been developed targeting genes such as *aflP*, *aflT*, *aflD*, and ITS regions for detecting aflatoxigenic *A. flavus* [30,106,116,117]. These assays demonstrate high sensitivity, with detection limits reaching picogram or femtogram levels [30].

Despite these advantages, LAMP assays may exhibit non-specific amplification and cross-reactivity with closely related species [115,116]. Additionally, false negatives can occur due to low DNA concentration or sample degradation [115]. Therefore, LAMP is most effective when used in combination with other molecular and analytical methods.

## 6. Recent Detection Techniques Mostly Focused on Aflatoxins Quantification but Limited to the Identification of Aflatoxigenic *A. flavus*

There are several methods already established for detecting AFs contamination in food and feed commodities, such as gas chromatography (GC), thin-layer chromatography (TLC), high-performance liquid chromatography (HPLC), fluorescence polarization assays, enzyme-linked immunosorbent assay (ELISA), and other microbiological methods used in a lab setting. These methods may provide accurate results; however, most of them need a well-organized laboratory, skilled personnel, and sophisticated methodologies [140]. Moreover, these techniques are usually expensive and need substantial time, particularly to detect AFs for large-scale screening, and are hard to incorporate into production or storage systems to monitor AFs detection or contamination. To minimize these limitations, some of the advanced techniques have been utilized to detect AFs contamination, which are rapid, sensitive, specific, and innovative.

### 6.1. Biosensor-Based Detections

A biosensor is a modern tool that is used to detect microorganisms and metabolites by merging biological recognition elements (DNA, enzymes, and antibodies) with a signal transducer. Those recognition elements usually bind to the specific target and develop a reaction, which is then converted through a transducer from a biological interaction to measurable signals (Electrical, Optical, and Physical). Biosensors have been widely used to detect mycotoxins in several foods and feeds. Depending on the bioreceptor and transducer, several types of biosensor systems have been utilized, focusing on different principles and functions such as aptamers, enzymes, nucleic acids, microorganisms, and protein-receptor based technique [141,142].

#### 6.1.1. Enzymatic Sensor

This type of biosensor mainly operates on the association between substrate detection and enzyme inhibition [143,144]. Enzymatic biosensors can be widely incorporated in different transducers such as calorimetric, optical, and electrochemical. However, the most commonly used transducer is electrochemical [145]. This type of biosensor has wide application in different fields such as food, agricultural, environmental, pharmaceutical, and medical industries [146]. One of the main reasons behind this is the rapid and cost-effective method along with its analytical performance, which is listed in the study of [147].

For example, this technique was performed for the detection of tyramine, which was cross-linked with glutaraldehyde inside calcium orthophosphate, and then an amperometric technique was performed to measure the changes in the electrochemical signal of o-dopaquinone. Also, citrinin mycotoxin in rice samples was measured through the use of an amperometric biosensor utilizing horseradish peroxidase [147,148]. Furthermore, several types of enzymes have been used in terms of enzymatic inhibition, such as urease, cholinesterase, and glucose oxidase. For example, a commonly used enzyme, Acetylcholinesterase (AChE), is used because of its susceptibility to mycotoxins. Another study also supported this statement that AFB1 was detected by the use of AchE enzymatic activity due to the inhibitory effect between AFB1 and AchE [149]. Moreover, another study revealed that the use of cholinesterase in the biosensor technique is useful for the detection of AFB1 [150]. However, the utilization of nanomaterials in the development of biosensors improves the performance metrics such as stability and sensitivity. The sensitivity can be improved by the methods of inhibition, enzyme amount, and incubation time. Due to these advantages, it could be adapted for on-site monitoring and large-scale screening of different compounds.

#### 6.1.2. Immunosensor

Immunosensors are termed as a bioanalytical device based on analyte-antibody interactions, where a specific antibody is used to detect a specific mycotoxin. In this technique, three primary groups have been developed, such as surface plasmon resonance, colorimetric sensors, and electrochemical sensors.

A label-free optical biosensor has been developed by Xu et al., (2013), which is based on gold nanorods and able to detect aflatoxin B1 [151]. The optical nano-biosensor provides highly selective, simple, and sensitive detection due to its strong ionic strength and reducing false results through the competitive dispersion principle [151]. AFB1 could be detected at a very low limit of detection (0.2 ng/mL) compared to the traditional ELISA method. For example, Surface plasmon resonance (SPR) is an optical detection technique that measures the total reflection of light upon a thin gold film, such as Au or Ag [152]. In one study, the sensitivity of the SPR was also higher compared to the ELISA method for AFB1 detection using the same immunoreagents. The sensitivity of the SPR was 3.0– 49 ng/mL, while the sensitivity of the ELISA was 12–25,000 ng/mL [153]. Also, electrochemical immunosensors are based on the principle of the competitive ELISA, in which an electrochemical transducer allows direct oxidation-reduction reactions [154]. The detection of AFB1 was observed through the electrochemical immunosensor, where the limit of detection was 0.15 ng/mL in a solution. Apart from this, this method is also utilized to detect OTA with higher specificity [155].

#### 6.1.3. Nucleic Acid Sensor or Aptasensors

Aptasensors are termed as biosensors, where aptamers (usually single-stranded RNA or DNA consisting of 2–60 nucleotides) particularly bind with the target molecule according to the lock-and-key theory [156,157]. Different immobilization strategies can influence aptasensor development, such as interactions between graphene oxide-modified interfaces and DNA base aptamers [158], binding thiolated aptamers to gold materials (Au-based materials) or CdTe quantum dots (QDs) [159], and linking aptamers to surface carboxylic groups [160].

So far, several aptasensors have been developed against different types of mycotoxins such as AFB1, OTA, fumonisin, ZEN, and T-2 toxin [158,161,162,163,164,165,166]. Moreover, mycotoxins, such as ZEN, OTA, and FB, in corn samples were detected based on multicolor quantum dot nanobeads with a detection limit of 5, 20, and 10 ng/mL, respectively [167]. An ultrasensitive RT-qPCR-based aptasensor has been developed that can detect AFB1 in foods and feeds with high sensitivity (25 fg/mL), specificity, and low cost [168]. Also, an enzyme-free sensor has been developed by Chen et al. to detect aflatoxin B1 by utilizing a catalytic DNA circuit. The circuit is developed by two components such as gold nanoparticles and three hairpin DNA probes, which recognize specific AFB1 [169]. The advantages of the aptasensors are that they can detect mycotoxin contamination through a high-throughput technique, which could be adapted in the field for large-scale on-site detection. Also, these aptasensors are low-cost, sensitive, portable, and rapid compared to the traditional techniques, HPLC-MS and HPLC with fluorescence detection (FLD). Despite several advantages, aptasensor-based detection has some limitations, such as the proper selection of aptamers, a time-consuming process, and distinguishing and identifying similar mycotoxins (AFB1, AFB2, AFM1, and so on). Therefore, more research is required for the development of a specific aptamer and a multi-mycotoxin analysis scheme. Even though a significant advancement has been developed, signal amplification techniques and aptamer probe stability should be increased.

#### 6.1.4. Solid-State Gas Sensor

It is another type of rapid and advanced technique that could be useful for the indirect detection of mycotoxins by utilizing volatile organic compounds (VOCs) emitted from the aflatoxigenic fungi [170,171]. Several aflatoxigenic species, such as *F. verticillioides*, *A. flavus*, and *P. verrucosum*, which release specific types of VOCs during their infection and colonization of different food and feed, could be utilized as reliable biomarkers for detecting the presence of mycotoxins [172].

The advantage of this method is that it provides a non-destructive, early method for detecting mycotoxins in different facilities, such as silos, storage facilities, and processing environments, offering an alternative to conventional analytical methods [49,173]. Also, mycotoxins can be detected even before significant toxin accumulation and visible fungal growth [171]. The main part of the principle of this technique is the interaction between the surface of the sensing layer and VOC molecules [174,175]. When aflatoxigenic fungi release VOCs, they interact with adsorbed O_2_ species on the surface of the sensing layer and ultimately release electrons. Then, the electrical signal is measured in proportion to the VOC concentration [176,177]. To improve the performance of the gas sensor, nanoparticles could be integrated, which increases the adsorption sites and surface area [178]. In addition, metal doping and specific coatings also increase the specificity of VOCs [179,180]. This technique is also associated with some limitations, such as interference from environmental factors and non-specific VOCs, which could interfere with the accuracy, reproducibility, and sensitivity of the detection [181].

### 6.2. Optical-Based Detections

Among the several techniques mentioned above, optical-based methods are a rapid and non-destructive method for AFs detection, which is based on fluorescence spectroscopy (FS), hyperspectral imaging (HSI), Raman spectroscopy, and near-infrared spectroscopy (NIRS).

#### 6.2.1. Fluorescence Spectroscopy (FS)

FS is termed an analytical technique that has been utilized in several disciplines to investigate the functions, structure, and reactivity of synthetic polymers, small molecules, other biological molecules, and proteins. Mainly, three processes are involved in the fluorescence process, such as excitation, conversion, and finally emission. The absorption of visible and ultraviolet light by a substrate or molecule is termed a fluorophore, which actually absorbs energy at a fixed wavelength and emits the light by liberating energy at a longer wavelength [182].

FS techniques have been widely used to detect mycotoxin contamination in several food and feed commodities [183]. There has been a link between AFs contamination, *A. flavus* infection, and bright-greenish-yellow fluorescence (BGYF), which was first reported in cottonseeds in 1969. After that, BGYF has been widely used as an indicator of AFs contamination [184]. A study performed by Wu et al. developed a self-built laser-induced fluorescence spectroscopy (LIFS) system integrated with three detection probes, which can classify AFB1-contaminated and non-contaminated pistachio kernels at a limit of detection of 5 μg kg^−1^ and a classification accuracy of more than 91.0% [185]. Previously, studies reported the detection of AFs through the traditional FS methods [184,186]; however, this method has some limitations, such as the production of kojic acids, which influences the fluorescence signal for AFs detection [187] and can lead to false positive results [188]. Apart from that, the FS method relies on one specific wavelength, which is utilized on the sample. Compared to this technique, a new technique has been developed, named Fluorescence Fingerprinting (FF), which is a method for detecting a specific emission pattern across several excitation wavelengths [189]. The FF method provides more sensitive and precise quantification compared to the FS [189], which is also a rapid and robust method of quantification of AFs [190].

#### 6.2.2. Raman Spectroscopy

Raman Spectroscopy is a non-destructive technique based on the scattered light incident, which interacts with the chemical bonds within a sample. The Raman spectra can be obtained by detecting the Raman shifts and it has been widely used for the detection of food-borne pathogens in both the food and agri-industries due to its rapid, specific, and sensitive method [191,192,193].

Several studies have performed Raman spectroscopy techniques to differentiate bacterial species from different sources with an accuracy ranging from approximately 87% to 100% [194,195,196]. It has also been useful to detect bacteria in different survival stages, such as the viable but non-culturable state and biofilm formation state, which are crucial in terms of food safety and public health issues [197,198]. Besides bacteria, this technique is also utilized in non-bacterial pathogens such as viruses and fungi [199,200]. Apart from detecting microbes, it is also useful for detecting hazards and mycotoxin contamination [201,202,203,204,205]. To minimize the limitations of the traditional Raman spectroscopy method, a new analytical technique has been developed utilizing new nanomaterials such as copper, silver and gold called surface-enhanced Raman spectroscopy (SERS) [206]. Due to its characteristic spectroscopic fingerprint and superfast data-processing technique, SERS is useful for the direct and rapid detection of mycotoxins. Even though several advancements have been made in the SERS technique [207,208,209], there are still limitations regarding large-scale application, utilization of proper and specific binding reagents, point of need screening, and improvement of the SERS data analysis. Also, due to the low probability and the low intensity of the Raman scattering, it is difficult to detect molecules that are below the ppm level [210].

#### 6.2.3. Near-Infrared Spectroscopy (NIRS)

Near-infrared spectroscopy (NIRS) relies on the interactions between the molecules in the samples and the absorbed light, which result in vibration and molecular overtone associated with several functional groups such as N-H, O-H, and C-H [211]. This technique has been widely used in the agricultural field, particularly as a promising approach for AFs detection and aflatoxigenic fungi due to low cost and high efficiency [212,213,214].

A diode array NIRS has been used in a study to separate moldy dried figs using spectroscopy and observed that the rate of separation between healthy and moldy figs was more than 88% by using partial least squares regression (PLSR) [214]. The classification accuracy can also be as high as 97% in terms of the detection of fungus infection [215]. To improve the detection technique of NIRS, a new technique has evolved named as Fourier transform near-infrared spectroscopy (FT-NIRS), which improves the accuracy, specificity, reproducibility, and wavelength resolution [216]. This improved method of detection can differentiate the contaminated and non-contaminated samples with an accuracy of more than 90% while utilizing a logarithmic linear classifier (LOGLC) and linear discriminant classifier (LDC) [217]. This accuracy rate was also further increased up to 100% with LDC to classify healthy and infected samples [218].

#### 6.2.4. Hyperspectral Imaging (HSI)

A hyperspectral imaging technique is a relatively new technique that combines imaging and spectroscopic methods to provide spatial and spectral information of the samples being tested [219]. Therefore, compared to the traditional spectroscopic technique, it provides chemical imaging of the samples, which is useful for fungal infection and the detection of AFs contamination. There are three approaches that are involved with this technique to acquire 3-D hypercubes, such as area scan, point scan, and line scan methods.

It is widely used in several fields such as agriculture, food safety, and quality [220,221,222,223,224,225]. One of the main advantages of this technique is its integration into a wide range of applications, such as total viable counts (TVC), Psychrotrophic plate counts (PPC), detection of bacteria, fungal infection, and microbial toxin detection [183]. Several studies have been reported to utilize this technique to determine the *Fusarium* and *Aspergillus-infected* cereal grains, along with measuring the level of AFs and fumonisin contamination [226,227,228,229,230]. Moreover, this method can also be adapted to monitor the early detection of mycotoxin contamination and fungal infection [231]. It has the potential to detect several microbial contaminants from different sources; however, it has some limitations. Some of the factors need to be evaluated further to enhance the efficacy and reproducibility of this method, such as minimizing redundant data during analysis, inaccuracy of the detection for homogeneous samples, and selecting the most effective wavelength. Moreover, integration of the exact reference value for the accuracy and effectiveness of the calibration model will improve better prediction.

## 7. Integration of Artificial Intelligence and Multi-Omics Approach in Aflatoxins Research

In the era of artificial intelligence, different advanced techniques have been integrated nowadays to monitor AFs risk prediction and AFs detection. For AFs detection, several ML-based models can be applied, such as Support Vector Machines, Random Forest, Gradient Boosting, and the classification of contamination risk using environmental datasets [232,233]. For example, Long Short-Term Memory (LSTM) and Convolutional Neural Networks (CNNs) can analyze time series and image-based datasets effectively up to 90% [234]. For the real-time prediction of AFs contamination, Hybrid AI-based models incorporate the factors related to fungal growth and colonization with sensor inputs, which will ultimately increase the prediction and interpretation [235,236]. Most of the machine learning models have a higher level of accuracy, ranging from 80 to 90% in terms of accurate classification of contaminated vs un-contaminated samples, forecasting contamination trends, and early contamination risk in storage conditions.

In addition, multi-omics-based approaches have also been developed recently in AFs research. In terms of metabolomics, several studies have been performed to identify the aflatoxigenic fungi, such as *A. flavus*, based on the chemical markers utilizing through gene expression [237,238,239]. Also, studies utilized metabolomics to determine the correlation between fungal infection and AFs accumulation [240,241]. Regarding the genomics approach, several genomics tools have been widely used for aflatoxigenic *A. flavus* [242,243,244] with a focus on whole-genome sequencing, AFs biosynthesis, and gene expression profiles [245,246,247]. Transcriptomics is one of the advanced fields in the recent era [248]. Transcriptomics research enables researchers to study gene expression at the transcriptional level along with gene expression regulation, functions of gene products, protein modification, and evolutionary changes [249,250]. For example, a study reported the use of RNA sequencing to identify gene functions related to *A. flavus* infection and AFs production [251,252]. Also, another study revealed how genes are expressed in different stages of infection, such as early or late stages [252]. Moreover, a transcriptomic study revealed how oxidative stress influences AFs accumulation [253]. Proteomics is another novel field that has been reviewed for AFs research [254]. Proteomic research in the mycotoxin field provides a clear understanding of cellular functions, protein profiling, and the effects of biotic factors. Also, it provides valuable information about plant responses against pathogen infestations and mycotoxin accumulation.

## 8. Conclusions and Future Perspectives

Over the years, both traditional (morphological and cultural) and molecular approaches have been developed for the detection of aflatoxigenic *A. flavus*. However, accurate identification remains challenging due to the complex nature of AFs biosynthesis, which is governed by multiple genes within the AFs biosynthesis gene cluster and influenced by gene expression dynamics and environmental conditions.

This review highlights that reliance on the presence of gene (single-gene or even multi-gene) identification strategies is often insufficient to definitively classify an isolate as aflatoxigenic. The presence or transcription of specific genes does not always correlate with actual AFs production, as toxin biosynthesis depends on a combination of genetic, physiological, and environmental factors. Therefore, not all *A. flavus* isolates possessing key biosynthetic genes can be considered aflatoxigenic.

**Figure 4:**
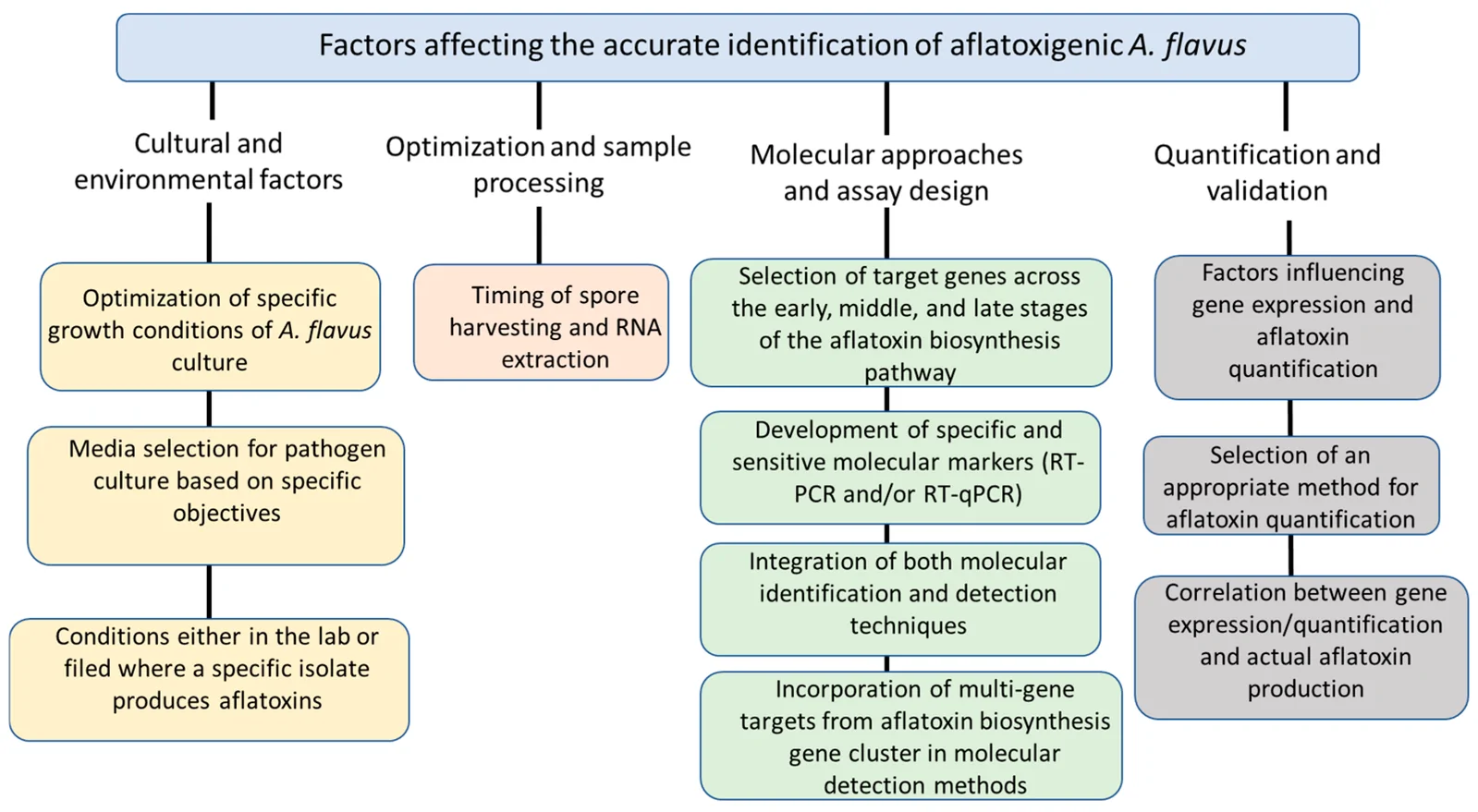
Key factors influencing accurate identification of aflatoxigenic *A. flavus*.

As illustrated in Figure 4, accurate identification requires careful consideration of multiple interacting factors, including the selection of appropriate molecular targets across different stages of the AFs biosynthesis pathway, optimization of gene expression analysis, proper culture conditions, and the integration of reliable quantification methods. Additionally, methodological factors such as the use of multi-gene detection strategies, timing of sample preparation, and selection of suitable media further influence detection accuracy.

**Figure 5:**
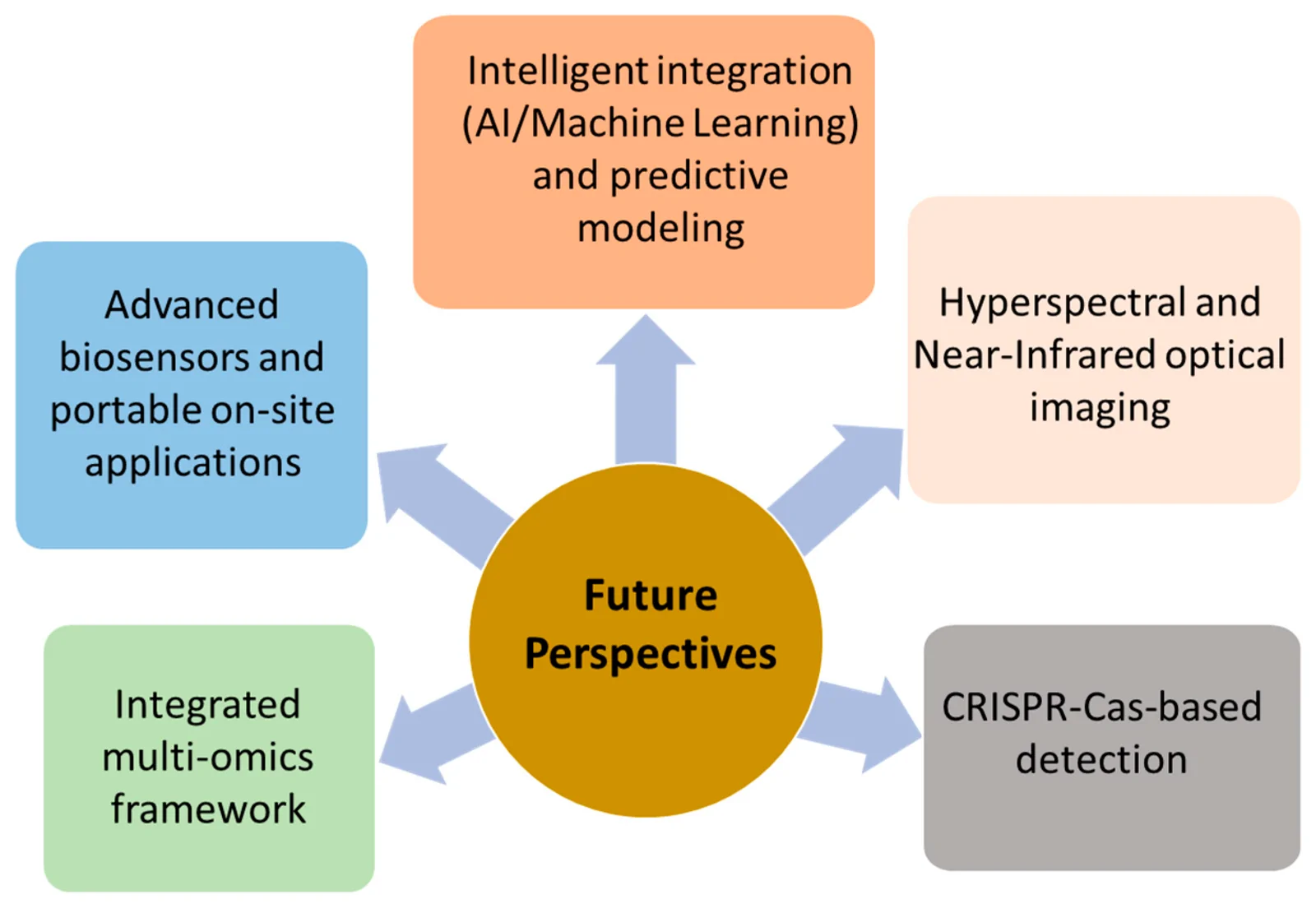
Future perspective on identification of aflatoxigenic *A. flavus* and AFs detection.

Looking forward, future research should focus on developing standardized, multi-target identification platforms that integrate molecular, biochemical, and environmental parameters along with advanced analytical or detection methods (Figure 4 and Figure 5). The incorporation of advanced molecular techniques, along with emerging tools such as biosensors and portable sequencing technologies, offers promising opportunities for rapid and on-site detection. Furthermore, integrating omics-based approaches, including genomics and transcriptomics, can improve the understanding of gene regulation and enhance the predictive accuracy of AFs production and aflatoxigenic *A. flavus* identification. With advances in machine learning and deep learning algorithms, techniques such as AI-based predictive modeling, CRISPR-Cas, HIS, and NIR could provide a framework for identifying aflatoxigenic isolates and assessing their AFs production.

In conclusion, the future of detecting aflatoxigenic *A. flavus* lies in the development of integrated, multidisciplinary strategies that align with the complex factors outlined in Figure 4 and the utilization of techniques depicted in Figure 5. Such approaches will significantly improve detection reliability and support effective food safety monitoring and sustainable disease management practices.

## Figures and Tables

**Table 3:** Comparison of different methods for accurate identification of aflatoxigenic *A. flavus*.

Methods/Techniques	Analysis Time	Cost	Sensitivity and Detection of Limit (LOD)	Specificity	Limitations	Field Applicability	Target Entity/Outcomes
Traditional/Cultural Techniques							
Ammonia Vapor Test	4–7 days	Low	Low		False negative	Low	Qualitative measurement of toxin production
Fluorescence-based detection	4–7 days	Moderate	Low		False negative	Low	Qualitative measurement of toxin production
Molecular Methods							
Standard PCR	3–5 h	Moderate to high	High	high	False positive/false negative	None	Only identify the presence of gene
RT-PCR	4–6 h	High	High	Very high	False negative	None	Determine gene expression of AFs genes
RT-qPCR	4–6 h	High	Very high	Very high	False positive/negative	None	Gene expression quantification of AFs genes
LAMP	30–60 min	Low to moderate	High	High	False positive/negative	High	Only identify the presence of gene
Analytical/Detection Methods							
Traditional (HPLC, LC-MS/MS, GC-MS, TLC, ELISA)	1–2 days	High to very high	Very high to excellent	Very high	False positive/negative	Low	Quantify Mycotoxins
Biosensor-based detection	15–45 min	Low	Excellent	Very high to excellent	False positive/negative	Very high	Quantify Mycotoxins

## Data Availability

No new data were created or analyzed in this study. Data sharing is not applicable to this article.

## Abbreviations

The following abbreviation is used in this manuscript:

AFs	Aflatoxins
